# The GLP-1 receptor agonist liraglutide inhibits necroptosis and neuroinflammation in a mouse model of Parkinson’s disease with diabetes co-morbidity

**DOI:** 10.3389/fnins.2025.1596506

**Published:** 2025-06-18

**Authors:** Xiaomin Zhang, Pengyang Du, Bo Bai, Peng Feng, Xia Lian, Christian Hölscher, Yongqing Wang, Guofang Xue

**Affiliations:** ^1^Department of Neurology, The Second Hospital of Shanxi Medical University, Taiyuan, China; ^2^Institute of Rehabilitation Medicine and Health Care, Henan Academy of Innovations in Medical Science, Zhengzhou, China

**Keywords:** Parkinson’s disease, diabetes mellitus, GLP-1, necroptosis, neuroinflammation

## Abstract

**Objective:**

To investigate the effects of the glucagon-like peptide-1 (GLP-1) receptor agonist liraglutide on motor function, necroptosis, and neuroinflammation in the brain in mice with diabetic Parkinson’s disease (PD) and its possible mechanism.

**Methods:**

We prepared a diabetic model with streptozotocin, followed by 1-methyl-4-phenyl-1,2,3,6-tetrahydropyridine-induced diabetic PD model, along with liraglutide or saline intervention, and the control group with equal volume saline. After that, body weight and blood glucose of each group mice were measured, and the open field experiment and gait analysis experiment were performed to detect the motor and non-motor symptoms of mice, western blotting and immunohistochemistry experiments were performed to test the levels of necroptosis-related proteins tumor necrosis factor-α (TNF-α), receptor interacting serine/threonine kinase 1 (RIP1) and neuroinflammation-related proteins nuclear factor kappa-B p-p65 (NF-κB p-p65), nuclear factor kappa-B p65 (NF-κB p65), interleukin-1 beta (IL-1β), and monocyte chemoattractant protein-1 (MCP-1).

**Results:**

The GLP-1 receptor agonist liraglutide inhibited necroptosis and neuroinflammation via TNF-α signaling in diabetic PD mice, decreasing blood glucose and improving motor function and mood. Compared with the control group, the blood glucose of diabetic PD mice increased, and the total distance and resting time in the central area decreased in the open field tests, stride length and overall run speed reduced in the gait analysis experiments, stance time and stride width increased, and the levels of necroptosis-related proteins such as TNF-α and RIP1, as well as neuroinflammation-related proteins including NF-κB p-p65/p65, IL-1β, and MCP-1 increased. Compared with the diabetic PD mice on the 7th day, the mice in the model group on the 21st day showed reduced motor activity and more severe brain injury. After administration of liraglutide, these impairments were reversed.

**Conclusion:**

In the diabetic PD model, liraglutide is neuroprotective by reducing necroptosis and neuroinflammation through the TNF-α signaling pathway.

## 1 Introduction

Parkinson’s disease (PD) is the second largest degenerative disease of the central nervous system in the world, with static tremor, myotonia, bradykinesia and postural disorder, affecting more than 1% of the elderly population (Lee and Gilbert, 2016). The pathological changes and clinical symptoms of PD show a progressive exacerbation trend, and this puts a huge burden on the patient’s family and society. At present, drug treatment is limited, and the body will have serious adverse reactions and complications with the extension of drug time (Prasad and Hung, 2021).

In recent years, the relationship between diabetes mellitus (DM) and PD has been gradually revealed. DM may be a risk factor for PD onset. The relative risk of PD is increased by 27% in patients with diabetes compared with non-diabetic patients (Aune et al., 2023). PD patients with DM had higher Unified-Parkinson Disease Rating Scale (UPDRS) motor scores and more severe Hoehn and Yahr stage compared to PD patients (Cereda et al., 2012). Therefore, diabetes aggravates the risk and progression of PD. The combination of these two common diseases can seriously affect the quality of life of patients. Understanding the molecular mechanisms of the disease helps to explore targeted therapeutic agents.

The pathogenetic mechanism of PD is complex, and neuroinflammation and necroptosis are the core processes (Iannielli et al., 2018; Oñate et al., 2020; Standaert, 2024). DM has a multifaceted impact on PD. In addition to the widely studied insulin dysregulation, necroptosis and neuroinflammation are also involved (Cheong et al., 2020; Sabari et al., 2023). Studies have shown that microglial cell necroptosis and neuroinflammation increased under high glucose stimulation, and the density of neurons in diabetic retina decreased, which aggravated retinal neurodegeneration (Huang et al., 2023). In the hippocampus of diabetic mice, tumor necrosis factor-α (TNF-α)—mediated necroptosis and nuclear factor kappa-B (NF-κB) pathway were activated, related inflammatory factors increased in neurons and glial cells. Application of necroptosis inhibitor Necrostatin reversed these changes, reduced amyloid aggregation in mice and improved cognitive function (Preeti et al., 2023). Therefore, necroptosis and neuroinflammation are crucial targets for preventing and treating DM, which is complicated with by neurodegenerative diseases such as PD.

Glucagon-like peptide 1 (GLP-1) is a 30-amino-acid incretin that functions in regulating insulin secretion, β-cell proliferation, cardiac and neurologic protection by combining the GLP-1 receptor (GLP-1R) in multiple systems of the body (Müller et al., 2019). Liraglutide, an acylated GLP-1 analog, is authorized for therapeutic management of DM and obesity (Tella and Rendell, 2015). The drug can cross the blood–brain barrier to activate GLP-1R in the brain, further regulating processes such as energy utilization, cell proliferation and differentiation, and inflammation (Hölscher, 2020; Hunter and Hölscher, 2012). In patients with polymyositis, GLP-1R agonists inhibited necroptosis through the AMP-activated protein kinase (AMPK) - phosphoglycerate mutase family 5 (PGAM5) pathway, thereby reducing muscle inflammation and ameliorating myasthenia symptoms (Kamiya et al., 2022). Studies have shown that liraglutide improves tissue damage by inhibiting necroptosis and inflammation. Zhou et al. (2023) found that liraglutide could reduce myocardial ischemia/reperfusion injury by decreasing necroptosis via the GLP-1R/phosphoinositide 3-kinase (PI3K)/protein kinase B (Akt) pathway. In addition, liraglutide alleviated neuroinflammatory response in PD and DM (An et al., 2023; Cao et al., 2022), preventing diabetic mice from reduced motor function and dopaminergic neurons loss, and reducing the risk of PD by up-regulating AMPK/ peroxisome proliferator-activated receptor γ coactivator 1a (PGC-1a) signaling (Ma et al., 2019). A phase 2 clinical trial showed some improvement in patients with PD (Hölscher, 2024). Another large clinical cohort study suggested that users of GLP-1R agonists and dipeptidyl peptidase 4 (DPP4) inhibitors had a 36–60% reduced prevalence of PD compared with diabetic individuals with other oral antidiabetic agents (Brauer et al., 2020). Previous research has indicated that liraglutide improved motor function, promoted repair and regeneration of synaptic or dopaminergic neuronal cells in PD models induced methyl-4-phenyl-1,2,3,6-tetrahydropyridine (MPTP) (Liu et al., 2015; Yuan et al., 2017). However, whether GLP-1 receptor agonists affect brain function in mice with diabetic PD through necroptosis and neuroinflammation remains unknown.

Here, we used liraglutide in the treatment of PD with diabetes co-morbidity mice to analyze whether liraglutide is protective in diabetic Parkinson’s disease mice, and to explore whether necroptosis and neuroinflammation are involved.

## 2 Methods

### 2.1 Experimental animals and groups

#### 2.1.1 Experimental animals

Healthy adult male C57BL/6J mice 8 weeks old (18–23 g), with SPF feeding conditions, were provided by the Experimental Animal Center of Shanxi Medical University. Mice were kept in a 12-h light–dark cycle with a mean ambient temperature of 22 ± 3°C and 50–55% relative humidity. Three mice per cage were provided with enough food and drink. To lower the experimental error caused by environmental maladaptation, mice were given an adaptation time of roughly 7 days before treatment.

Ethics statement: The Second Hospital Ethics Committee of Shanxi Medical University authorized experimental plans used in this study, permission no. 2022L156. The ARRIVE guidelines were adhered to at all times.

#### 2.1.2 Groups of experimental animals

Firstly, after 7 days of adaptation to the animal housing, 12 mice were randomly divided into four groups (*n* = 3 in each group): (A) the control group (CON group, *n* = 3); (B) the 7-day group of diabetes model (D-7D group, *n* = 3); (C) the 7-day group of Parkinson’s disease model (P-7D group, *n* = 3); (D) the 7-day group of diabetic Parkinson’s disease model (DP-7D group, *n* = 3).

**Figure 1:**
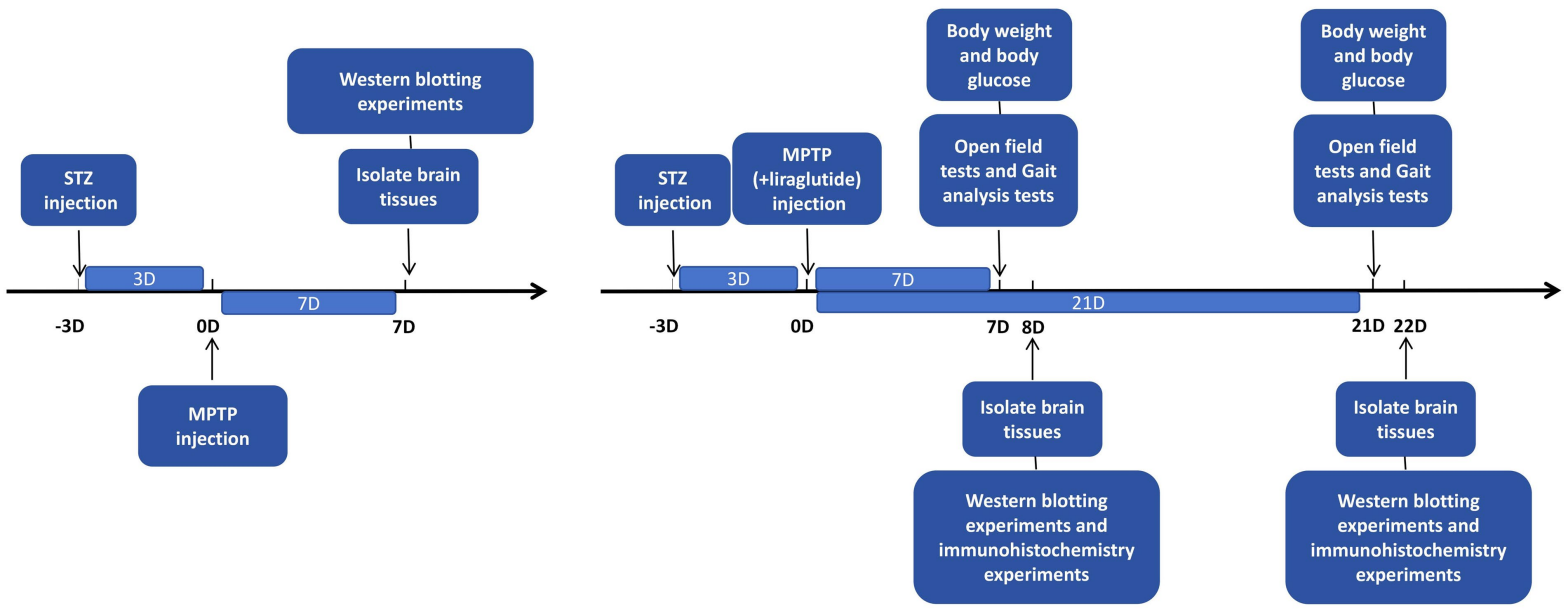
Schematic representation of the experimental flow.

Secondly, 60 mice were randomly divided into five groups (*n* = 12 in each group), all mice except the control group completed the diabetes model making first: (A) the control group (CON group, *n* = 12): normal saline (0.2 mL/d, once, intraperitoneal injection, 21 days); (B) the 7-day group of diabetic Parkinson’s disease model (DP-7D group, *n* = 12): MPTP (25 mg/kg/d, once, intraperitoneal injection, 7 days) + normal saline (0.2 mL/d, once, intraperitoneal injection, 7 days); (C) the 7-day group of treatment with liraglutide (LIR-7D group, *n* = 12): MPTP (25 mg/kg/d, once, intraperitoneal injection, 7 days) + liraglutide (25 nmol/kg/d, once, intraperitoneal injection, 7 days); (D) the 21-day group of diabetic Parkinson’s disease model (DP-21D group, *n* = 12): MPTP (25 mg/kg/d, once, intraperitoneal injection, 7 days) + normal saline (0.2 mL/d, once, intraperitoneal injection, 21 days); (E) the 21-day group of treatment with liraglutide (LIR-21D group, *n* = 12): MPTP (25 mg/kg/d, once, intraperitoneal injection, 7 days) + liraglutide (25 nmol/kg/d, once, intraperitoneal injection, 21 days) (Figure 1).

### 2.2 Induction and drug treatment of Parkinson’s disease with diabetes co-morbidity model

Streptozotocin (STZ) was purchased from Sigma (United States). Prepare citric acid buffer solution freshly, adjust pH = 4.2–4.5, then dissolve STZ in 1% buffer, the configuration and injection process avoid light and in a cold bath, completing the injection within 30 min, intraperitoneal injection. MPTP was purchased from MCE (China), and the drug was dissolved in a concentration of 25 mg/kg in 0.9% saline and injected intraperitoneally. Liraglutide injection was purchased from Novo Nordisk (Denmark), and the drug was dissolved in 0.9% saline at a final concentration of 25 nmol/kg and injected intraperitoneally.

Diabetic modeling: After 7 days of adaptive feeding, mice were given 80 mg/kg STZ after 8 h of fasting for 3 days. After 72 h of adequate diet, we collected tail vein blood collection to measure random blood glucose, which was greater than 16.8mmo/L, namely diabetes induction was successful.

Parkinson’s disease animal model preparation: MPTP 25 mg/kg/d administered intraperitoneally for 7 days.

The DP-21D group mice were given an equal volume of saline 30 min after MPTP injection, for 21 days. The LIR-7D group mice were treated with liraglutide 25 nmol/kg at 30 min after MPTP injection, for 7 consecutive days. The LIR-21D group mice were treated with liraglutide for 21 days; The CON group mice received daily intraperitoneal injection of equal volume of normal saline. On days 7 and 21 after treatment, mice of each group were sacrificed under anesthesia by cervical dislocation method. Among them, 2 mice in DP-21D group died during MPTP administration, and we supplemented them in time to ensure sufficient sample size.

### 2.3 Blood glucose and body weight testing

Blood glucose and body weight of mice were measured on days 1, 3, 7, 10, 14, 17, and 21 after liraglutide or normal saline treatment. Blood glucose measurement method: tail vein blood sampling and values recorded with a glucometer.

### 2.4 Behavioral experiments

#### 2.4.1 The open field experiment

The experiment device contained a 40 cm*40 cm*40 cm square open field, and the bottom was divided into 16 equal size squares according to the method of 4*4, calling the middle four grids as the central zone. The infrared camera detected the center of gravity of the mice and recorded the moving trajectory, which was analyzed by the computer Smart 3.0 system. Mice adapted overnight in the laboratory, the following day, they were positioned in the same spot in the center of the open field, and their locomotor activity was recorded for the next 10 min. During the experiment, avoid the light, and avoid the mice seeing the experimenter, and do the barrier protection. After testing each mouse, the open field area was wiped with 75% alcohol and dust-free paper to prevent urine, feces, and odor effects on the activity of the next mouse. The open field experiments were performed by an investigator who was blinded to this experiment. We analyzed the total distance and resting time in the central area (Note: Resting time in the central area: the total time spent in the central zone).

#### 2.4.2 Gait analysis experiment

The experiment required mice to freely pass the 115 cm*6.5 cm colorless transparent glass runway three times in the dark, and Bcam Capture software was used to gather mouse footprints on a 30 cm-long runway, and the Runway Scan 3.0 plate gait analysis system was used to analyze the footprints. For testing, mice were placed in the dark environment of the laboratory for one night and tested the next day. For testing, mice were put on the track to pass it, ensuring that they had to pass continuously through the set 30 cm long glass track in one direction, mice were required to perform in three consecutive sessions. For parameter statistics, the RunwayScan3.0 system accurately recognized and labeled 11 consecutive footprints per mouse. The statistical analysis did not include data from mice who stopped or turned throughout the trial. We analyzed the right foot average (FR Avg) stride length, stride width, FR Avg stance time, and overall Avg run speed (Note: Stride Length: The length of each step of the mouse can be used to reflect pace stability and basic activity ability. Stride Width: Track width is the distance between the midpoint of the left forefoot standing track and the midpoint of the right forefoot standing track. It is the two front feet distance apart. This index reflects the motor balance and coordination. Stance Time: The time the limbs touched the ground during walking. This index reflects the supporting ability and exercise endurance of the mouse limbs).

### 2.5 Sample preparation and testing

#### 2.5.1 Isolation and fixation of the mouse midbrain tissue

Each mouse group was randomly split into two sections following the behavioral assessments. The first section was used for the western blotting experiments. Mice (*n* = 6 per subgroup) were narcotized with 4% chloral hydrate (5 mL/kg, intraperitoneal injection), the corneal reflex of mice disappeared after 5–10 min, then mice were perfused with 200 mL of normal saline. After treating mice with cervical dislocation method, the midbrain tissue was separated on ice and stored in the −80°C refrigerator. The second part was used for the immunohistochemical staining. After treating the mice with the above anesthetic method, cardiac perfusion was performed with 100 mL of normal saline and 100 mL of 4% paraformaldehyde (PFA) solution to fix tissue. Rapid cervical dislocation treatment was administered to the mice, followed by the removal of the brains, which were preserved in 4% PFA solution for a whole day and the part of the substantia nigra tissue was cut out. We used an automated biological tissue dehydrator for gradient dehydration, and then embedded the samples after paraffin, sectioned and stained them.

#### 2.5.2 Western blotting experiments

Fresh mouse midbrain tissue was weighed and mashed in EP tubes, and RIPA lysate containing PMSF and phosphatase inhibitor in a 10:1 ratio lysed fully the tissue. The above operation was performed on ice. The solution was further centrifuged at 13,000 rpm/min for 15 min and the supernatant was retained. The bicinchoninic acid (BCA) technique was used to quantify the protein concentration and proteins of each group were adjusted to the same concentration with loading buffer and radio immunoprecipitation assay (RIPA) lysate, boiled for 10 min until the protein denaturation, then packed and stored in −80°C refrigerator. After dispensing with the sodium dodecyl sulfate - polyacrylamide gel electrophoresis (SDS-PAGE) gel preparation kit, an equal amount of 20 μL protein sample and 5 μL protein Marker were loaded, adjusting the voltage at 80 V and constant voltage electrophoresis for 2.5 h to separate proteins. After electrophoresis, the glue was separated, the polyvinylidene fluoride (PVDF) membrane was activated in methanol for 1 min, and the membrane “sandwich” was prepared. Put into the membrane clip in the order of “filter paper - glue - transfer printing film - filter paper,” placed into the membrane tank, filled the membrane buffer, added crushed ice around the membrane tank, constant pressure 80 V for 1.5 h. After the membrane transfer, the membrane was washed with TBST, and then the PVDF film was dipped into the blocking solution, and was left for 1 h on the shaker. Then, the following rabbit anti-mouse primary antibody plus antibody dilution to make the antibody working solution: β-actin (1:1000), TH (1:500), α-syn (1:1000), TNF-α (1:500), RIP1 (1:500), NF-κB p-p65 (1:500), NF-κB p65 (1:500), IL-1β (1:1000), and MCP-1 (1:500) polyclonal antibody, and the PVDF film was dipped into the working solution in a 4°C refrigerator overnight. The next day, following TBST washing, the secondary antibody working solution was prepared from goat anti-rabbit IgG-HRP secondary antibody (1:5000) diluted with antibody dilution, then immersed in PVDF membrane, and incubated for 1 h at room temperature in a shaker. The PVDF membrane was incubated with the secondary antibody, followed by another TBST wash, staining, and treated with chemiluminescence (ECL) reagent. The optical density values of the target bands were analyzed by the Image J software. During data processing, the ratios of the target protein to the internal standand β-actin in each sample were all normalized to the corresponding ratios of the control group. The magnitude of the ratio reflects the relative level of the protein.

#### 2.5.3 Immunohistochemical experiments

Mouse substantia nigra was isolated and fixed in 4% PFA for 24 h. The tissue was dehydrated by an automatic dehydrator, and cut into 5 μm slices with a semi-automatic slicer and attached to glass slides. After drying for 2 h, slices were dewaxed in xylene solution, absolute ethanol, 95% ethanol, 85% ethanol, 75% ethanol, and distilled water. Following a 15-min incubation period at room temperature in 3% H_2_O_2_, the slices were washed in PBS. Then, the slice rack was placed into boiling antigen repair solution, heated continuously heated for 10 min, and then cooled naturally to room temperature. Then the racks were submerged in PBS. The following rabbit anti-mouse primary antibodies were applied to slices: β-actin (1:100), TH (1:100), α-syn (1:100), TNF-α (1:100), RIP1 (1:100), NF-κB p65 (1:100), NF-κBp-p65 (1:100), IL-1β (1:100), MCP-1 (1:100) for 4°C overnight, after that, we added the HRP-labeled goat anti-mouse secondary antibody to the slices at 37°C for 1 h. After antibody incubation, 100 μL of ready-made DAB solution was added to show the color, and we immediately stopped the reaction in water when the color just deepens. Then the slices were soaked in hematoxylin for 3 min to counterdye, slowly rinsed with tap water, then immersed in 1% hydrochloride ethanol for 3 s, and immediately immersed in tap water and running water blue for 20 min. After returning, the slices were successively immersed in 75% ethanol, 85% ethanol, 95% ethanol, absolute ethanol, xylene, and finally sealed with neutral resin. Under a microscope, optical density analysis was carried out, and the average optical density (AOD) values for positive staining within a specific region were measured and used for statistical analysis. A higher AOD value indicates a relatively higher expression of the target antigen in the region, and a lower AOD value indicates a lower antigen expression and shallower staining. These, respectively, correspond to higher or lower protein levels. Cells with brown granules were considered immunoreaction-positive. The magnification is 400×. The software Image-Pro Plus 6.0 was used to analyze the cells.

#### 2.5.4 Statistical methods

GraphPad Prism 8.0.2 was used to analyze the experimental data. Blood glucose and body weight data were analyzed using two-factor repeated measures ANOVA with duplicate data, the remaining data were analyzed by one-way ANOVA, and the Tukey’s *post-hoc* test was used to compare groups of data. The threshold for statistical significance was set at *p* < 0.05. Results are presented as the means ± SEM.

## 3 Results

### 3.1 The mouse model of Parkinson’s disease with diabetes co-morbidity

**Figure 2:**
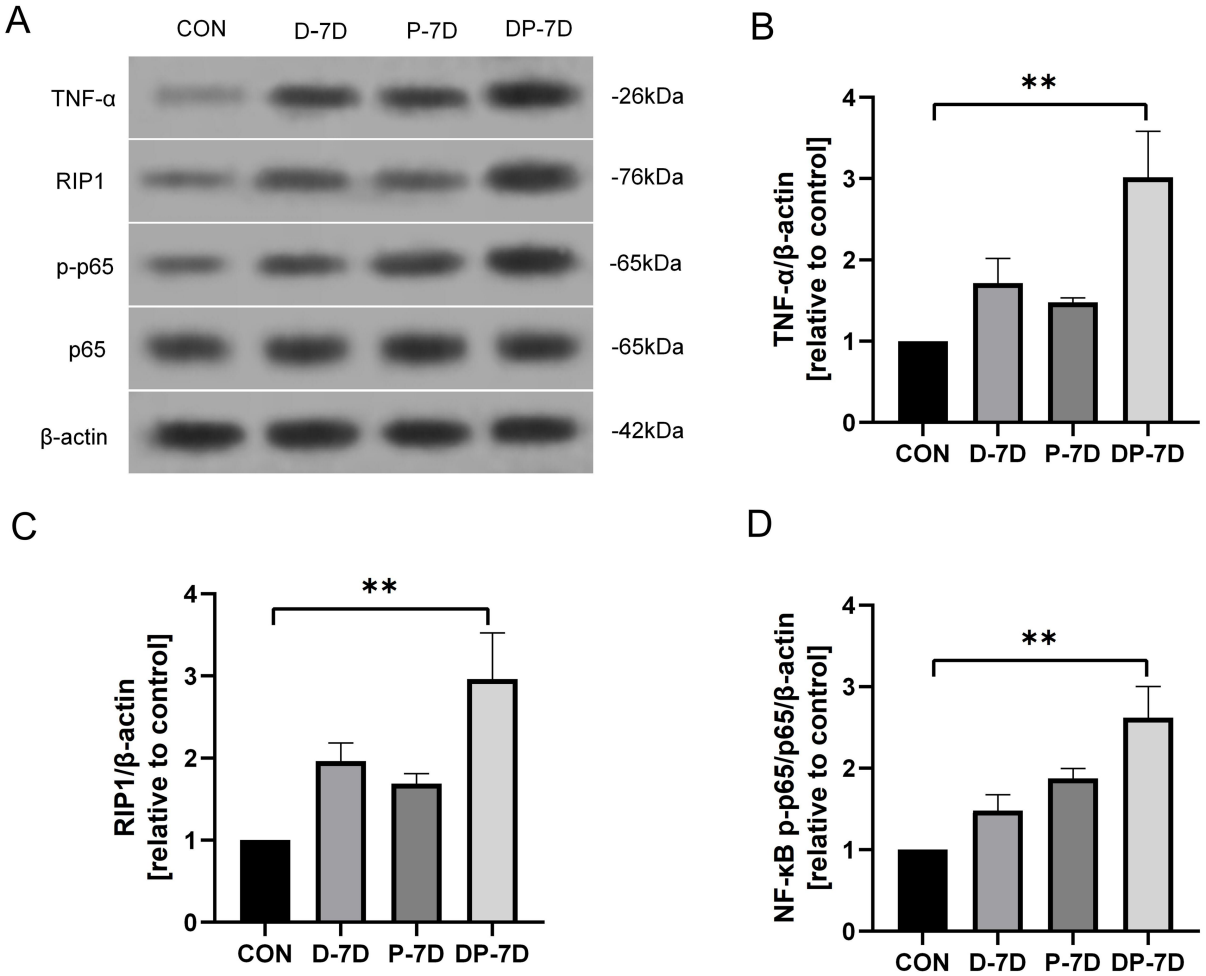
Levels of tumor necrosis factor-α (TNF-α), receptor interacting serine/threonine kinase 1 (RIP1) and nuclear factor kappa-B (NF-κB) p-p65/p65 levels of the mice in each group. **(A)** Western blotting bands of TNF-α, RIP1 and NF-κB p-p65/p65. **(B–D)** Quantitation of TNF-α, RIP1 and NF-κB p-p65/p65. *p < 0.05; **p < 0.01; ***p < 0.001; *n* = 3.

First, we detected the levels of TNF-α, receptor-interacting serine/threonine kinase 1 (RIP1), and NF-κB p-p65/p65 levels in the midbrain of mice using western blotting experiments. The results showed that compared to the CON group, the DP-7D group had elevated TNF-α levels (*p* < 0.01, Figures 2A,B), RIP1 levels (*p* < 0.01, Figures 2A,C) and NF-κB p-p65/p65 levels (*p* < 0.01, Figures 2A,D). The D-7D and the P-7D group also showed an increasing trend in these indicators compared to the CON group, but without statistical significance.

The results indicated that the PD with diabetes co-morbidity mice model had more severe pathological damage, so this dual-disease model was used for the following experiments.

### 3.2 Blood glucose and body weight of mice

**Figure 3:**
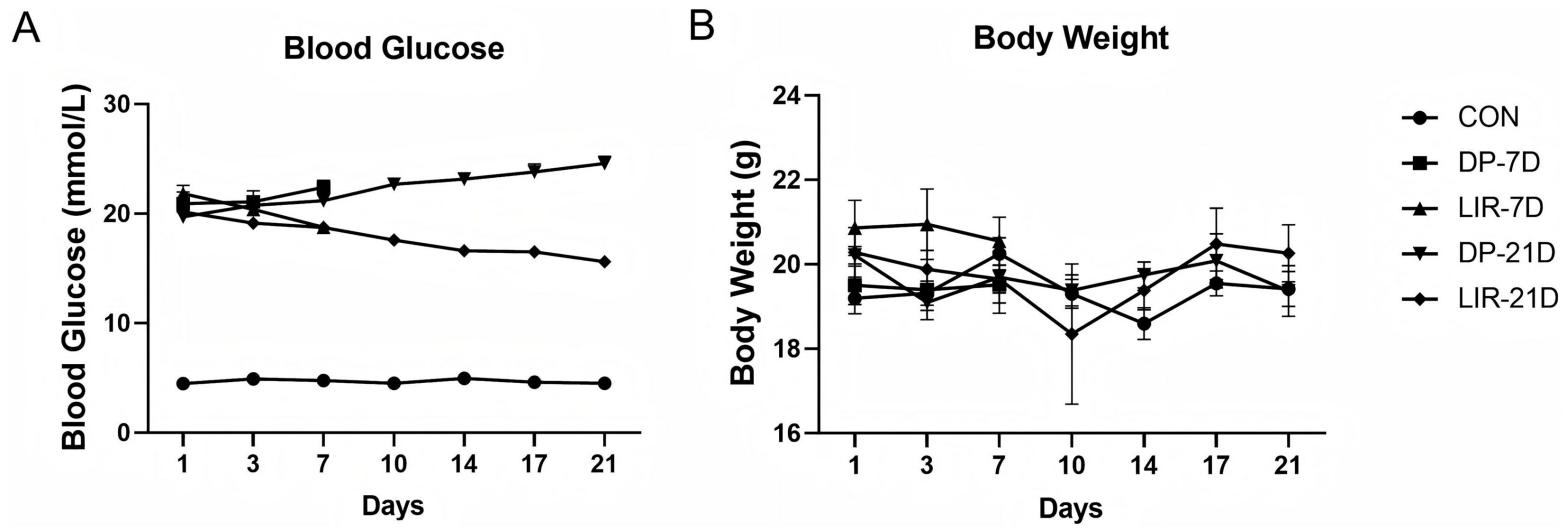
Blood glucose and body weight levels of the mice in each group. **(A)** Blood glucose of mice was monitored on days 1, 3, 7, 10, 14, 17, and 21 after liraglutide or normal saline treatment. **(B)** The mice’s body weight was measured on days 1, 3, 7, 10, 14, 17, and 21 after liraglutide or normal saline treatment. *n* = 6.

The mice in the other four groups had elevated blood glucose at all time points in contrast to the control mice (*p* < 0.001, Figure 3A). Compared to the DP-7D group, the blood glucose levels of the LIR-7D group mice dropped on day 7 (*p* < 0.01, Figure 3A), and compared with DP-21D group, blood glucose decreased in LIR-21D group (*p* < 0.01, Figure 3A). On the following 10, 14, 17, and 21 days, compared to the DP-21D group, the LIR-21D group experienced a greater drop in blood glucose (*p* < 0.001, Figure 3A), suggesting that liraglutide treatment reduced blood glucose levels in diabetic mice with PD, and had no effect on blood glucose in the non-diabetic control mice. When analyzing body weight on days 1, 4, 7, 10, 14, 17, and 21, no difference between the groups was detected (*p* > 0.05, Figure 3B).

### 3.3 Motor and non-motor activity of mice

#### 3.3.1 The open field experiment

**Figure 4:**
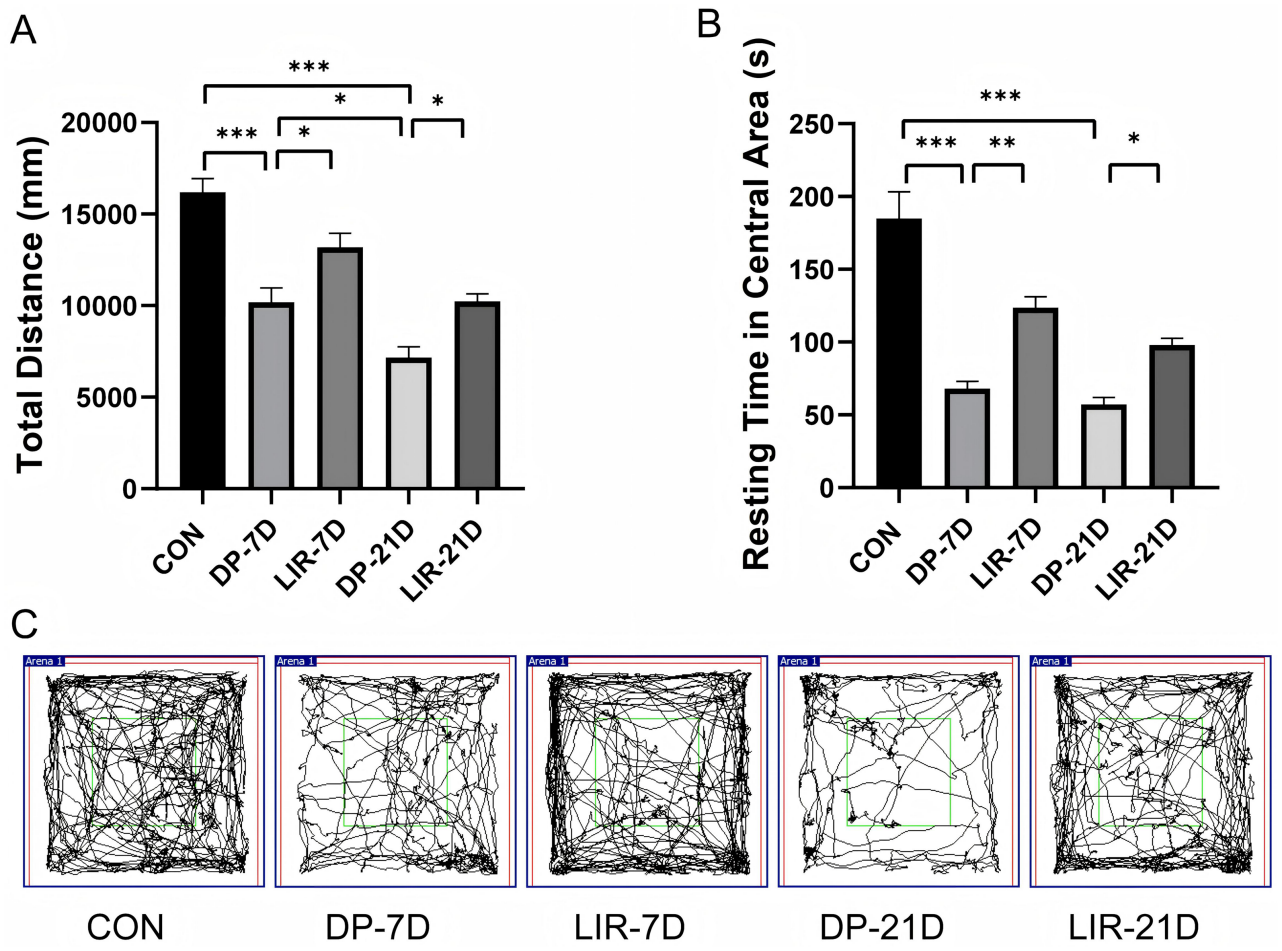
Activity quantification and representative trajectories of each group in the open field experiment. **(A)** Quantification of total distance in each group. **(B)** Quantification of resting time in central area in each group. **(C)** Representative tracks in the open field test of each group. *p < 0.05; **p < 0.01; ***p < 0.001; *n* = 6.

The total distance and resting time in central area decreased in DP-7D and DP-21D groups in contrast to the control group (*p* < 0.001, Figure 4), which suggested decreased motor function and increased anxiety in model groups. Liraglutide reversed the above changes. Total distances and resting time in the central area increased in LIR-7D mice as compared to the DP-7D group (*p* < 0.05, Figure 4A and *P* < 0.01, Figure 4B). In comparison to the DP-21D group, the LIR-21D group experienced an increase in total distance and resting time in the central area (*p* < 0.05, Figures 4A,B). In addition, between the two model groups, the total distance of DP-21D mice was decreased (*p* < 0.05, Figure 4A).

#### 3.3.2 Gait analysis experiment

**Figure 5:**
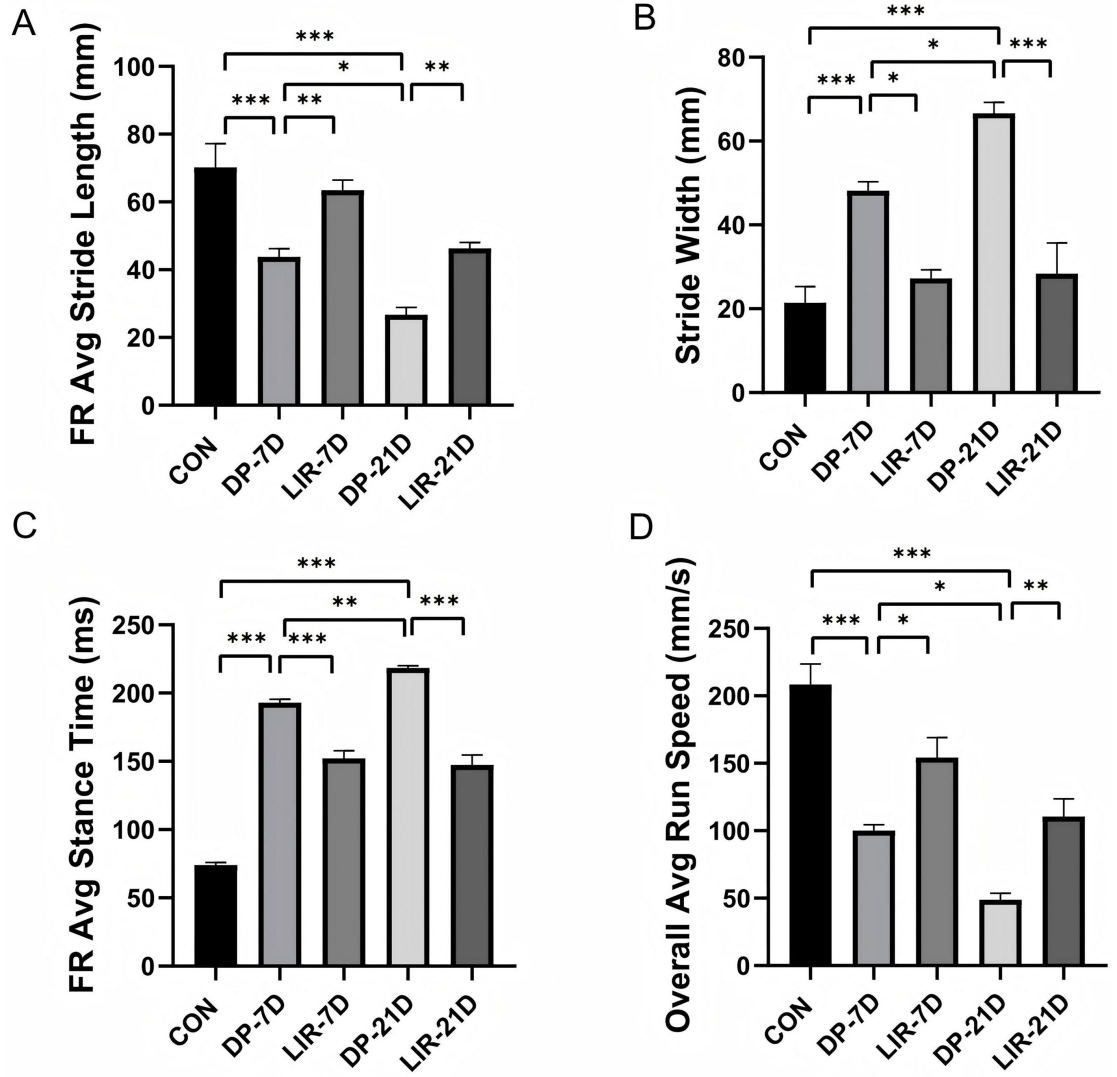
Quantification of behavioral activity in each group of mice in the gait analysis experiment. **(A)** Quantification of right foot average (FR avg) stride length in each group. **(B)** Quantification of stride width in each group. **(C)** Quantification of FR avg. stance time in each group. **(D)** Quantification of the overall avg. run speed in each group of mice. *p < 0.05; **p < 0.01; ***p < 0.001; *n* = 6.

DP-7D and DP-21D groups had decreased FR avg. stride length and overall avg. run speed, and increased stride width and FR avg. stance time compared with control mice (*p* < 0.001, Figures 5A–D), suggesting worse motor coordination, gait instability, and slower movement speed in diabetic PD mice. Liraglutide improved motor function in diabetic PD mice.

Compared with the DP-7D group, LIR-7D mice had increased FR avg. stride length (*p* < 0.01, Figure 5A) and overall avg. run speed (*p* < 0.05, Figure 5D), and decreased stride width (*p* < 0.05, Figure 5B) and FR avg. stance time (*p* < 0.001, Figure 5C). Compared with the DP-21D group, LIR-21D mice had increased FR avg. stride length (*p* < 0.01, Figure 5A), shorter stride width (*p* < 0.001, Figure 5B), shorter FR avg. stance time (*p* < 0.001, Figure 5C), and faster overall avg. run speed (*p* < 0.01, Figure 5D). In addition, we found that DP-21D mice had smaller FR avg. stride length, increased stride width than DP-7D group (*p* < 0.05, Figures 5A,B), increased FR avg. stance time (*p* < 0.01, Figure 5C), and decreased overall avg. run speed (*p* < 0.05, Figure 5D).

### 3.4 Dopamine neurons and α-synuclein protein content changes in the substantia nigra of mice

**Figure 6:**
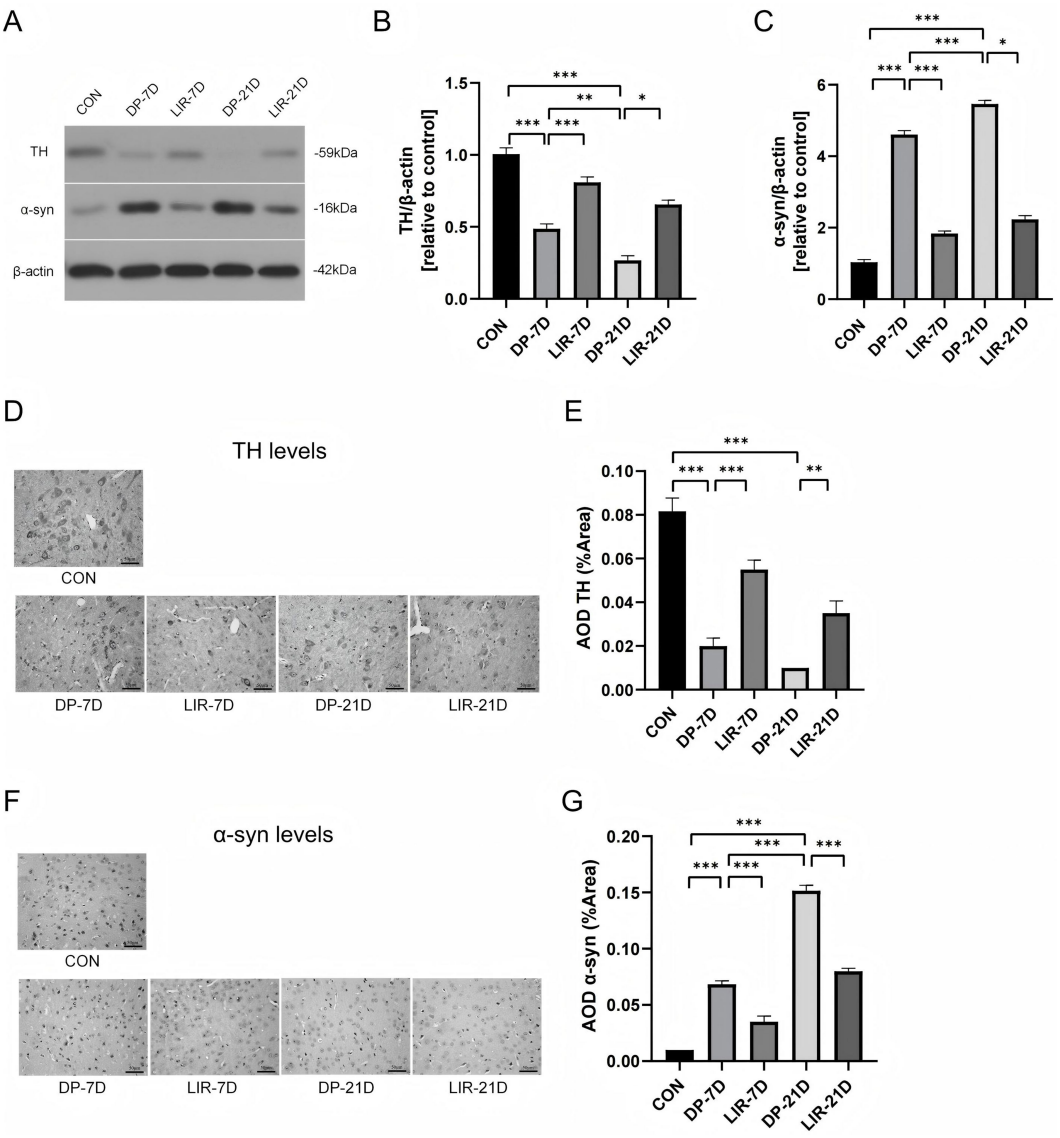
Tyrosine hydroxylase (TH) and α-synuclein (α-syn) levels in each group’s mice midbrain. **(A–C)** Western blotting bands and quantitation of TH and α-syn. **(D,E)** Immunohistochemical images and quantification of TH. **(F,G)** Immunohistochemical images and quantification of α-syn. Scale bar = 50 μm. *p < 0.05; **p < 0.01; ***p < 0.001; *n* = 6.

We examined the levels of the key dopamine neuron enzyme Tyrosine hydroxylase (TH) and α-synuclein (α-syn) in the mouse substantiae nigrae using western blotting experiments and immunohistochemistry experiments (Figure 6).

In the western blotting experiments, we found that TH levels decreased in DP-7D and DP-21D groups (*p* < 0.001, Figures 6A,B), and liraglutide significantly increased the TH levels (*p* < 0.001, DP-7D vs. LIR-7D; *p* < 0.05, DP-21D vs. LIR-21D, Figures 6A,B). A-syn levels increased significantly in DP-7D and DP-21D groups (*p* < 0.001, Figures 6A,C), and in contrast, liraglutide significantly lowered α-syn levels (*p* < 0.001, DP-7D vs. LIR-7D; *p* < 0.05, DP-21D vs. LIR-21D, Figures 6A,C).

We detected differences in TH and α-syn levels between the groups by immunohistochemical experiments (Figures 6D–G). For TH, when comparing the DP-7D and DP-21D mice to the control mice, the levels of TH dropped (*p* < 0.001, Figures 6D,E), and liraglutide medication reversed it. For α-syn, the levels of α-syn were significantly increased in DP-7D and DP-21D mice in contrast to control groups (*p* < 0.001, Figures 6F,G), and the above changes were reversed by liraglutide treatment.

The DP-21D group exhibited higher α-syn levels (*p* < 0.001, Figures 6A,C,F,G) and decreased TH levels in comparison to the DP-7D group (*p* < 0.01, Figures 6A,B).

### 3.5 TNF-α/RIP1 signaling and necroptosis of mice substantia nigra

We used western blotting experiments and immunohistochemical experiments to examine TNF-α and RIP1 levels in mouse substantiae nigrae.

**Figure 7:**
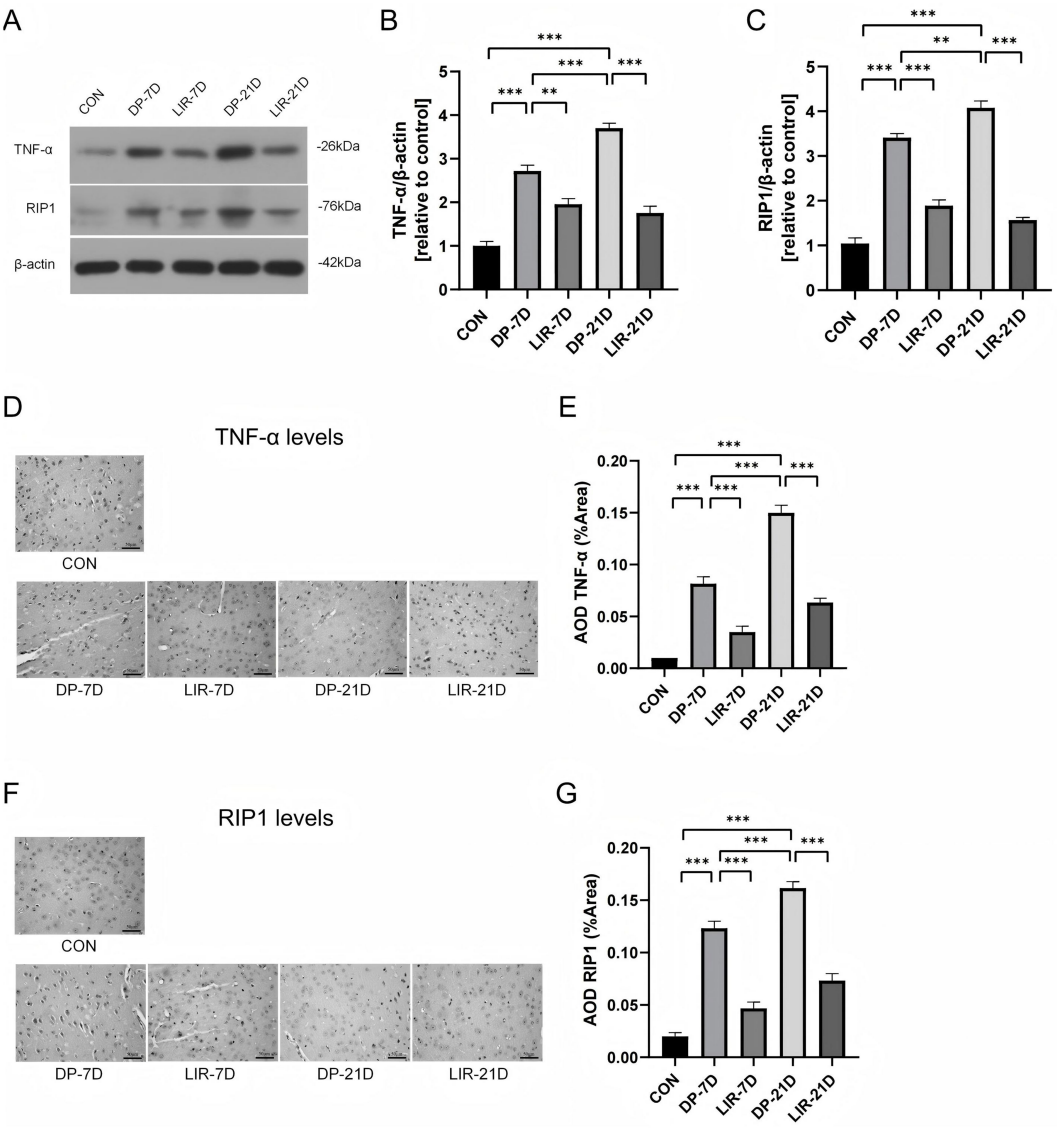
Levels of tumor necrosis factor-α (TNF-α) and receptor interacting serine/threonine kinase 1 (RIP1) in the necroptotic pathways in the brain of mice in each group. **(A–C)** Western blotting bands and quantitation of TNF-α and RIP1. **(D,E)** Immunohistochemical images and quantification of TNF-α. **(F,G)** Immunohistochemical images and quantification of RIP1. Scale bar = 50 μm. *p < 0.05; **p < 0.01; ***p < 0.001; *n* = 6.

In western blotting experiments, it was showed that TNF-α and RIP1 increased in the midbrain of DP-7D group and DP-21D group (*p* < 0.001, Figures 7A–C), TNF-α decreased in the LIR-7D and LIR-21D group compared with the model group (*p* < 0.01, DP-7D vs. LIR-7D; *p* < 0.001, DP-21D vs. LIR-21D, Figures 7A,B), and RIP1 decreased significantly in the LIR-7D and LIR-21D group compared with the corresponding model group (*p* < 0.001, Figures 7A,C). In addition, the DP-21D group exhibited a considerable rise in TNF-α and RIP1 when in contrast to the DP-7D group (*p* < 0.001, Figures 7A,B; *p* < 0.01, Figures 7A,C).

In immunohistochemistry experiments, we found that significantly increased TNF-α and RIP1 levels in the model mouse compared with control mice (*p* < 0.001, Figures 7D–G) and TNF-α and RIP1 decreased after liraglutide treatment (*p* < 0.001, Figures 7D–G). TNF-α and RIP1 levels decreased in DP-21D group compared with DP-7D group (*p* < 0.001, Figures 7D–G).

### 3.6 The NF-κB signaling pathway of mice substantia nigra

We found differences in NF-κB, interleukin-1 beta (IL-1β), and monocyte chemoattractant protein-1 (MCP-1) levels between the groups by western blotting and immunohistochemistry experiments.

**Figure 8:**
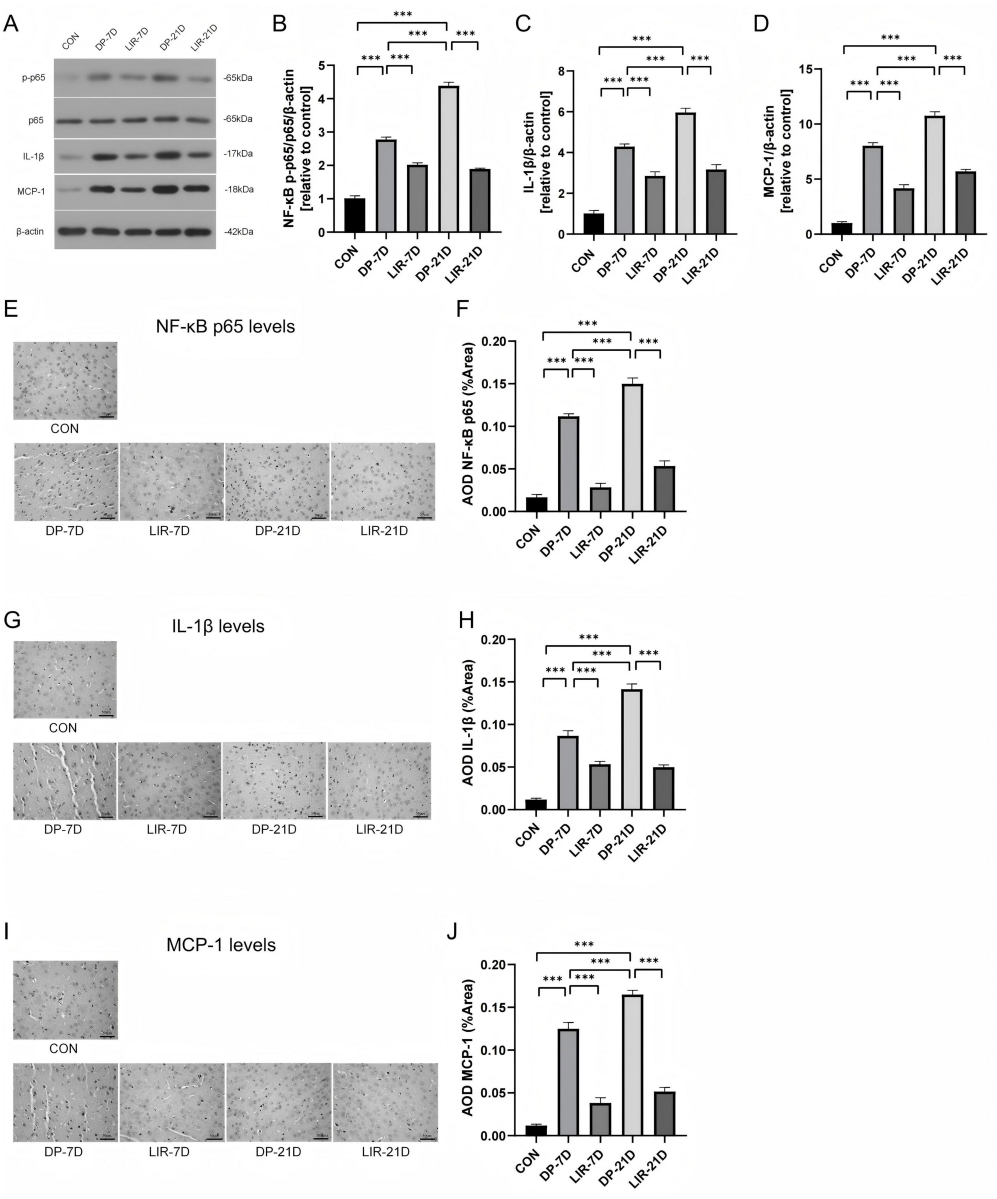
Levels of nuclear factor kappa-B (NF-κB) pathway-related molecules NF-κB p-p65/p65, interleukin-1 beta (IL-1β), and monocyte chemoattractant protein-1 (MCP-1) in the brain of each group of mice. **(A–D)** Western blotting bands and quantification of NF-κB p-p65, NF-κB p65, IL-1β, and MCP-1. **(E,F)** Immunohistochemical images and quantification of NF-κB p65. **(G,H)** Immunohistochemical images and quantification of IL-1β. **(I,J)** Immunohistochemical images and quantification of MCP-1. Scale bar = 50 μm. *p < 0.05; **p < 0.01; ***p < 0.001; *n* = 6.

Overall, the model mice’s substantiae nigrae had higher amounts of NF-κB, IL-1β, and MCP-1, which suggested that the NF-κB signaling pathway had been activated, and liraglutide treatment reduced the above molecular levels, indicating that liraglutide inhibited the NF-κB signaling pathway. Specifically, in western blotting experiments, the results indicated in the midbrains of DP-7D and DP-21D mice, NF-κB p-p65/p65, IL-1β and MCP-1 levels were significantly higher than control animals (*p* < 0.001, Figures 8A–D), and following liraglutide therapy, NF-κBp-p65/p65, IL-1β, and MCP-1 levels were lower (*p* < 0.001, Figures 8A–D). Compared with DP-7D, the DP-21D group showed a rise in NF-κBp-p65/p65, IL-1β, and MCP-1 (*p* < 0.001, Figures 8A–D).

In the immunohistochemical experiments, we found significantly increased NF-κB p65, IL-1β and MCP-1 in DP-7D and DP-21D substantiae nigrae (*p* < 0.001, Figures 8E–J), the levels of NF-κB p65, IL-1β, and MCP-1 were significantly decreased in LIR-7D and LIR-21D group mice after liraglutide treatment (*p* < 0.001, Figures 8E–J). In addition, we found that the above three indicators were increased in DP-21D group compared with DP-7D group mice (*p* < 0.001, Figures 8E–J).

## 4 Discussion

Our study revealed that liraglutide is neuroprotective in mice with diabetic PD. The results of this study demonstrated that liraglutide slowed the pathological damage and improved motor deficits and mood abnormalities of mice with diabetic PD by inhibiting TNF-α-mediated necroptosis and neuroinflammation. Studies have shown that in the open field test, the total distance and resting time in the central area of diabetic PD mice induced by streptozotocin (STZ) and MPTP decreased. In the gait analysis test, the stride length reduced, the stride width and stance time increased, and the running speed decreased. In addition, compared with the control group, the content of the pathological protein α-syn in the brains of diabetic PD mice increased, the content of TH decreased, the expressions of necroptosis-related proteins TNF-α and RIP1 increased, and the levels of NF-κB p-p65/p65, IL-1β, and MCP-1 in the NF-κB inflammatory pathway increased.

Liraglutide can cross the blood–brain barrier and directly bind to the GLP-1R in nerve cells, activating multiple pathways such as the cyclic adenosine monophosphate (cAMP) pathway and the phosphatidylinositol 3-kinase (PI3K) pathway. It exerts effects such as promoting synaptic repair, improving amyloid protein pathology, blocking inflammation and reducing oxidative stress. All these processes are independent of glucose (Hölscher, 2020; Hunter and Hölscher, 2012). Our previous studies have also shown that multiple GLP-1R agonists can play neuroprotective role in diabetic rats with cerebral ischemia–reperfusion injury, PD mice, status epilepticus rats and other disease models without relying on blood glucose (Bai et al., 2021; Lv et al., 2021; Wang et al., 2018). Our study has also confirmed this point. Liraglutide directly exerted a neuroprotective effect by inhibiting the TNF-α-mediated RIP1 necroptosis and the neuroinflammatory pathway. This suggests that liraglutide may have multiple protective mechanisms when used in diabetic PD.

Typical symptoms of PD include motor symptoms and non-motor symptoms such as mood abnormalities. Gait analysis experiment evaluates the gait function and limb coordination of mice through multiple parameters, which is an important behavioral research method in PD (Goldberg et al., 2011). In our study, mice of the model groups showed decreased stride length and overall run speed, and increased stride width and stance time, indicating increased gait instability and decreased limb coordination, which are consistent with the movement symptoms of PD patients, liraglutide improved the above movement disorders. This may be related to the recovery of dopaminergic neuron death, the details are as follows. Open field experiment is often used in animal studies of emotional abnormalities such as anxiety, providing information on motor ability and anxiety-related emotional behaviors. Under the high-pressure environment, animals are more inclined to avoid the exposed central area, stay in the area near the wall, their exploring behavior reduce, and show anxiety (Horka et al., 2023). In this study, resting time in the central area of diabetic PD mice decreased, indicating increased anxiety mood and decreased total distance indicating decreased locomotor activity. Liraglutide improved the anxiety-like behavior and motor function of the mice.

The main pathological features of PD are the degenerative death of dopaminergic neurons in the substantia nigra and the formation of Lewy bodies containing abnormally aggregated α-syn fibrils. Aggregation and transmission of α-syn leads to dopaminergic neuron loss and dopamine levels reduction of mice, triggering Parkinson-like neurodegeneration and being a key biomarker of Parkinson’s disease (Luk et al., 2012). TH is a key enzyme in dopamine synthesis and is an important tool to quantify the damage and loss of dopaminergic neurons. Roostalu et al. (2019) used combined immunohistochemistry with whole-brain 3D image reproduction method and found a significant loss of TH positive neurons in the substantia nigra of MPTP-induced PD mice, which showed a correlation with impaired motor coordination in mice. Consistent with this, we found decreased TH levels and increased α-syn in diabetic PD mice substantiae nigrae, and liraglutide treatment reversed these changes.

Necroptosis is a regulated type of cell death that is activated by the key protein RIP1, it is involved in the pathogenic mechanisms of various neurodegenerative diseases, such as PD, Alzheimer’s disease, amyotrophic lateral sclerosis, multiple system atrophy, etc. (Yuan et al., 2019). TNF-α is the best characterized inducer of necroptosis, playing a role in mediating the cell death and inflammatory (Oñate et al., 2020). When cells are stimulated by pathogens, TNF receptors are activated and this causes RIP1 activation, which further phosphorylates receptor-interacting protein kinase 3 (RIP3) and downstream mixed lineage kinase domain-like (MLKL), resulting in the formation of the necrosome, causing the destruction of cell membrane leading to necroptosis (Bertheloot et al., 2021; Conrad et al., 2016). In recent years, necroptosis has become an important therapeutic target in neurodegenerative diseases (Yuan et al., 2019). Increased RIP1 in microglia, mitochondrial, and lysosomal defects in neurons induce together the development of necroptosis. Studies have demonstrated that RIP1, RIP3 and pMLKL expression increased in brain tissue of PD patients (Hu et al., 2019; Oñate et al., 2020), and necroptosis pathway was activated in 6-hydroxydopamine (6-OHDA)-treated mice and midbrain primary neurons. The use of RIP1 inhibitors reduces axonal degeneration and loss of neurons, and improves motor activity in mice (Oñate et al., 2020). Our experiments showed that TNF-α and RIP1 levels elevated in diabetic PD mice substantiae nigrae and the necroptosis pathway was activated, while liraglutide reversed these changes and further ameliorating the histopathological defects in substantiae nigrae. Liraglutide can be neuroprotective by reducing TNF-α-mediated necroptosis.

In neurodegenerative diseases, necroptosis promotes the activation of the inflammatory pathway and downstream inflammatory factors, forming an inflammatory environment and further aggravating nerve cell damage. Inflammatory mediators such as TNF-α also mediate the activation of necroptotic pathway, thus forming a vicious circle and aggravating the disease progression (Yuan et al., 2019). The necroptosis initiating molecule RIP1 has also become a key mediator of inducing neuroinflammation (Kim et al., 2023; Yuan et al., 2019). Kim et al. (2023) found that application of the RIP1 inhibitor Necrostatin-1 blocked the necroptosis pathway and pro-inflammatory cytokines TNF-α and IL-1β production, and upregulated the expression of neurotrophic factors and restored dopaminergic neurons in PD mice. Therefore, we examined the inflammatory markers associated with the NF-κB pathway to explore whether TNF-α-induced necroptosis further induced neuroinflammation in diabetic PD mice substantiae nigrae.

Neuroinflammation is an important factor in progressive neurodegeneration in PD, and this process involves several signaling pathways, among which the NF-κB pathway is crucial in the inflammatory progression of in PD (Singh et al., 2020). In physiological situations, NF-κB exists as a dimeric complex and binds to the IκB protein and thus is in an inactive state. When inflammatory factors and antigen receptors are bound to cell surface receptors, IκB kinase β (IKKβ) phosphorylates IκB and is degraded by the proteasome. Released and translocated into the nucleus, NF-κB binds to the DNA promoter and promote the transcription of related genes such as IL-1β and MCP-1. There is evidence that the concentrations of TNF-α, IL-1β, MCP-1, IL-2, and IL-6 increased significantly in the peripheral blood of PD patients (Qin et al., 2016; Santaella et al., 2020), inflammatory factor administration of IL-1β aggravated DA neuron loss in PD rats (Pott Godoy et al., 2008). MCP-1 was positively correlated with PD progression (Santaella et al., 2020). Our previous study showed that GLP-1R agonists played a neuroprotective role by decreasing NF-κB and increasing glial cell-derived neurotrophic factor (GDNF) expression in MPTP-induced PD mice (Liu et al., 2015; Lv et al., 2021; Yuan et al., 2017). Studies have shown that Polydatin suppresses the NF-κB pathway by activating protein kinase B (AKT)/glycogen synthase kinase-3β (GSK3β)-nuclear respiratory factor 2 (Nrf2) route, reducing loss of dopaminergic neurons in PD rats (Huang et al., 2018). Our study observed that NF-κB p-p65/p65, IL-1β, MCP-1 elevated in mice substantiae nigrae in the model group, indicating the activation of this pathway. However, liraglutide reversed the above-mentioned changes.

In order to observe the progression of the disease, we established diabetic PD models and treatment groups at 7 days and 21 days, respectively. Among them, compared with the 7 days model group, the mice in the 21 days model group showed more significant motor impairment and anxiety. The expression of TH in the brain decreased, while the expression of α-syn increased. The levels of necroptosis related proteins TNF-α, RIP1, and neuroinflammation related proteins NF-κB, IL-1β, and MCP-1 were higher. The results indicated that the pathological process of diabetic PD is time-dependent, providing a crucial time window for the early intervention of the comorbidity of diabetes and PD.

In conclusion, our experiment showed that liraglutide exerted a neuroprotective effect by inhibiting the necroptosis and neuroinflammatory response mediated by the TNF-α pathway, reducing the histopathological damage in diabetic PD mice, and improving their motor impairment and emotional state. The use of this drug in diabetic PD patients has direct neuroprotective benefits, prompting us to focus on liraglutide in basic research and clinical practice and actively apply it in cases of this co-morbidity. The application of this drug may have enormous social benefits by delaying the disease progression and thus improving the life quality of the patients. Several clinical trials in PD patients showed good effects testing GLP-1 receptor agonists. A first phase 2 clinical trial testing Liraglutide in non-diabetic PD patients showed limited effects, due to the fact that the main motor test readout had been obscured by a placebo effect in the control group (Hölscher, 2024), so further clinical trials are needed to confirm the medication’s therapeutic efficacy in diabetic people with PD.

## Data Availability

The raw data supporting the conclusions of this article will be made available by the authors, without undue reservation.
